# Repetitive hypoxic preconditioning protects retinal ganglion cells against damage caused by exposure to blast

**DOI:** 10.1371/journal.pone.0349124

**Published:** 2026-06-08

**Authors:** Matthew M. Harper, Nickolas A. Boehme

**Affiliations:** 1 Veterans Administration Center for the Prevention and Treatment of Visual Loss, Iowa City VA Healthcare System, Iowa City, Iowa, United States of America; 2 Department of Ophthalmology and Visual Sciences, The University of Iowa, Iowa City, Iowa, United States of America; 3 Department of Biology, The University of Iowa, Iowa City, Iowa, United States of America; Northumbria University, UNITED KINGDOM OF GREAT BRITAIN AND NORTHERN IRELAND

## Abstract

Visual system damage and dysfunction caused by exposure to a blast wave has been described in both clinical studies and in pre-clinical models. Within the retina, retinal ganglion cells (RGC) exhibit sensitivity to mild blast-mediated traumatic brain injury (bTBI), which can result in progressive neurodegeneration. The purpose of this study was to determine if repetitive hypoxic preconditioning (HPC) can prevent bTBI-mediated RGC damage and death. This study utilized clinically relevant outcomes of RGC structure and function, supported by histological analysis of the surviving RGCs. Mice were exposed to six sessions of HPC over a two-week period at an 11% oxygen concentration, and subsequently subjected to bTBI using a shock tube. Four-weeks following exposure to bTBI or sham, functional and structural analysis of RGCs was performed using the pattern electroretinogram (PERG) and optical coherence tomography (OCT). BRN3A antibody labeling was subsequently used to quantify the number of RGCs surviving at the termination of the study. Analysis of RGC outcomes showed significantly decreased PERG amplitude and RGC Complex + retinal nerve fiber layer (RNFL) thickness in mice with bTBI compared to sham. There was no significant difference in RGC outcomes between sham mice and HPC+ bTBI mice. Taken together, these results show that HPC can provide at least partial neuroprotection to RGCs prior to blast exposure.

## Introduction

Traumatic brain injury caused by exposure to a blast wave (bTBI) is increasingly recognized as a cause of persistent, and sometimes progressive, visual pathway deficits and retinal dysfunction [1,2]. Within the retina, retinal ganglion cells (RGCs) exhibit particular susceptibility to injury produced by blast exposure [3,4]. Acute injury to RGCs after bTBI is caused both from primary mechanical damage in addition to secondary vascular and metabolic changes that affect both the RGC soma and also axons within the optic nerve. Multiple secondary cascades that include oxidative stress, upregulation of inflammatory and neurotoxic molecules, mitochondrial dysregulation, and activation of the immune system have been described that could further amplify RGC loss [5–10]. Consistent with these observations, injury severity and cumulative blast exposure influence the timing and magnitude of RGC dysfunction and loss [1,11].

One emerging theme is that RGC fate after blast exposure may be modifiable by interventions that engage endogenous stress-resistance pathways. We previously reported that repeated low-intensity (35 kPa) blast exposures before a single high-intensity (137 kPa) blast exposure reduced RGC loss compared with a single 137 kPa injury [12]. Furthermore, we showed that these preconditioning blast injuries could prevent the upregulation of molecules that are implicated in RGC death. These findings have suggested that sub-injurious conditioning stimuli distinct from blast may also increase retinal resilience prior to bTBI.

In our current study we sought to determine if hypoxic preconditioning (HPC) prior to blast exposure could prevent blast-mediated RGC damage, similar to blast-preconditioning. HPC involves exposure to environments with decreased oxygen concentrations, which induces upregulation of neuroprotective molecules, most often related to hypoxia-inducible factor 1 (HIF-1) [13,14]. In the retina, HPC has been shown to protect RGCs from ischemic, excitotoxic, and mechanical damage, and has been associated with improved cellular function and reduced pro-death signaling [15–18]. Despite robust evidence supporting HPC in various injury models [19], its neuroprotective potential in blast-induced retinal trauma remains unexplored.

Understanding how HPC modulates the structural and functional integrity of RGCs after blast exposure may provide critical insight into the cellular pathways that promote retinal resilience. The present study investigates whether HPC confers neuroprotection to RGCs when administered prior to experimental bTBI and examines the functional and structural RGC response. Here we use three orthogonal retinal outcomes to quantify RGC vulnerability and test whether HPC preserves RGC function and structure after blast exposure. Our outcomes include: pattern electroretinography (PERG) to assess RGC-dependent function, optical coherence tomography (OCT) to measure in vivo structural integrity of the ganglion cell complex, and histologic RGC quantification to determine neuronal survival at endpoint. Together, these complementary readouts provide a rigorous evaluation of whether pre-injury HPC can shift the trajectory of blast-induced optic neuropathy toward a more resilient phenotype.

## Materials and methods

### Animals

All animal studies were conducted in accordance with the ARVO Statement for the Use of Animals in Ophthalmic and Vision Research and were approved by the Iowa City Department of Veterans Affairs Institutional Animal Care and Use Committee (Protocol 2091201). Male C57BL/6J (Stock 000664) mice were purchased from The Jackson Laboratory and enrolled in the study at 8 weeks of age. A total of 45 mice were used in this study.

### Hypoxic preconditioning

For this study 3 cohorts of C57Bl6/J mice were enrolled. Mice were enrolled in the study in the following groups: 1) healthy control mice exposed to sham blast (sham), 2) mice not receiving hypoxia treatment and exposed to blast (bTBI), and 3) mice receiving hypoxia treatment and exposed to blast (bTBI+ HPC). Hypoxic treatment for mice occurred 6 times over a two-week period prior to bTBI at 11% oxygen for 2 hours per exposure in a BioSpherix ProOx model P360 (Parish, NY), using compressed nitrogen to displace the oxygen in the chamber. Mice not receiving HPC were placed in the same room as HPC mice, but were not exposed to hypoxia.

### Blast injury

Two days following the final HPC treatment anesthetized mice were exposed to three 206 kPa blast waves, with each blast separated by a period of one day. Blast exposure was produced using an advanced blast simulator (ABS, Stumptown Research and Development, Black Mountain, NC) in which a compressed-gas driven system generates a blast wave, consistent with the general geometry of ABS platforms used in prior studies. The ABS test section measured 30 cm × 30 cm, and animals were positioned 1.93 m from the driver. Blast waves were initiated by rupture of a 26.5 oz coated fabric membrane (Mehler Technologies, Hückelhoven, Germany) fixed between the driver and expansion sections of the device. The total distance from the driver to the distal end of the blast-wave eliminator was 4.11 m.

Before exposure, mice were anesthetized with ketamine (30 mg/kg, IP) and xylazine (5 mg/kg, IP) and secured in restraint with the head facing the direction of the incoming wave. Sham animals underwent identical anesthesia and placement within the ABS but were not subjected to blast. Following bTBI, mice recovered on a warming pad. Anesthesia was reversed with yohimbine chloride (1 mg/kg, IP). After recovery, both sham and blast-exposed animals received buprenorphine analgesia (0.05 mg/kg, subcutaneous) immediately post-blast.

### Pattern evoked electroretinography

RGC function was quantified using PERG by measuring the amplitude of the evoked waveform. Mice were anesthetized with ketamine (30 mg/kg, IP), xylazine (5 mg/kg, IP), and acepromazine (2 mg/kg, IP), and placed on a heated platform. Responses were elicited using alternating, reversing black-and-white gratings presented on an LED display (Jorvec, Miami, FL). A subdermal recording electrode was positioned in the snout equidistant from both eyes. A reference electrode was inserted at the base of the head and a ground electrode at the base of the tail. PERG recording parameters were consistent with previously described methods [9,10,24,28,31–33].

To reduce placement-related variability, each mouse was placed 10 cm from the stimulus monitor. The PERG stimulus parameters were: 18° radius visual angle (full-field), 1.5 cm × 14 cm bars, 2 reversals/s, 372 sweeps averaged, band-pass filter 1–30 Hz, 98% contrast, and 80 cd/m^2^ mean luminance. Recordings were obtained under mesopic ambient conditions (8.5 lux) without dark adaptation using luminance matched reversals. The PERG outcome included the waveform amplitude (peak to trough) [32,34]. Only left-eye data are reported. Prior to PERG, mice were examined by handheld slit lamp to verify absence of anterior segment injury.

### Spectral domain optical coherence tomography

Retinal structure was evaluated using a Spectralis SD-OCT (Heidelberg Engineering, Vista, CA) configured with a 25D lens for mouse imaging [32]. Mice were anesthetized (ketamine 30 mg/kg IP; xylazine 5 mg/kg IP) and maintained on a warming pad. Pupils were dilated with 1% tropicamide and the cornea was kept hydrated with saline. Volume scans were acquired centered on the optic nerve head using a 49-line dense scan (15 A-scans/B-scan) over a 20° scan angle with a 20° × 25° scan field. The primary structural outcome was thickness of the RGC complex plus the retinal nerve fiber layer (RNFL), defined as RNFL + ganglion cell layer (GCL) + inner plexiform layer (IPL). A single B-scan from the superior retina, approximately 150 µm from the peripapillary region, was selected and quantified by an examiner masked to treatment group.

### Immunohistochemical quantification of RGCs

Four weeks following the final blast exposure mice were euthanized using CO_2_ followed by cervical dislocation, and eyes were enucleated. Posterior eyecups were isolated and fixed in 4% paraformaldehyde for 4 hours. RGCs were labeled using BRN3A immunohistochemistry following published procedures [36]. In brief, posterior cups were incubated overnight at 37°C in 0.3% Triton X-100 in PBS (PBST). Retinas were dissected and depigmented using 3% hydrogen peroxide in 1% sodium phosphate buffer for 3 hours at room temperature. Tissue was further permeabilized by incubation in PBST for 15 minutes at −80°C, then blocked overnight in 2% normal donkey serum in PBST.

Primary labeling was performed with anti-BRN3A (1:200; MAB1585, Millipore) prepared in 2% normal donkey serum with 1% Triton X-100 and 1% DMSO, incubated at 4°C for two nights. Samples were then incubated with secondary antibody (1:200; Alexa Fluor 488 donkey anti-goat, Invitrogen) for 4 hours at room temperature, counterstained with TO-PRO-3 iodide (1:1000, Molecular Probes), mounted on glass slides, flat-mounted with ProLong Diamond Antifade Mountant (Fisher Scientific), and coverslipped.

Flat mounts were imaged by confocal microscopy (Nikon AX R) at 400 × . For each retina, four non-overlapping central fields (1024 × 1024 px; 0.18 mm^2^) were captured adjacent to the optic nerve head (see Hedberg-Buenz et al. for field placement [36]); each field included a 3–5 plane z-stack. ImageJ was used for processing: maximum-intensity z-projection, background subtraction (rolling ball radius 35 px), and smoothing. Images were binarized using Huang thresholding, then refined with Open, Watershed, and Fill Holes operations. BRN3A^+^ nuclei were enumerated using Analyze Particles with size limits of 20–150 µm^2^ and circularity 0–1.

### Statistical analysis

Data are reported as mean ± standard deviation. All analyses were performed by investigators masked to group assignment. Distribution normality was assessed using the D’Agostino–Pearson omnibus K2 test. Comparison of groups was performed using One-way ANOVA. Statistical analyses were conducted in GraphPad Prism (v.10; GraphPad Software). Raw data are available at ODC-TBI.org.

## Results

**Fig 1:**
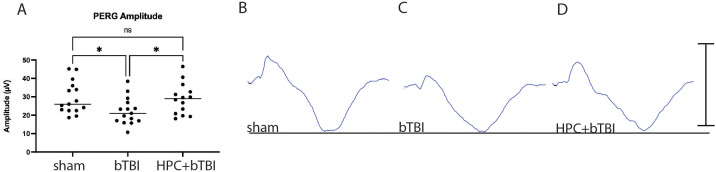
HPC preserves RGC function following bTBI compared to mice not exposed to HPC. The PERG amplitude of bTBI mice was significantly lower compared to sham mice (A, B, C, p = 0.001) and mice exposed to HPC prior to bTBI (D, p = 0.03). There was not a difference in the PERG response when sham and HPC+ bTBI groups were compared (A, D, p = 0.83). (* = p < 0.05). Scale Bar in D = 40 µV. One-way ANOVA with Fisher’s LSD post-test.

The PERG amplitude in mice exposed to sham bTBI was 29.47 ± 8.79 µV four weeks following sham exposure (Fig 1A, 1B). bTBI mice (Fig 1A, 1C) had a PERG response of 22.18 ± 7.31 µV, which was significantly lower compared to sham mice (p = 0.019). There was a also significant difference between bTBI and bTBI+ HPC mice (28.82 ± 8.42 µV, p = 0.035, Fig 1A, 1D). However, there was not a significant difference in the PERG amplitudes of sham mice and bTBI+ HPC mice (p = 0.83).

**Fig 2:**
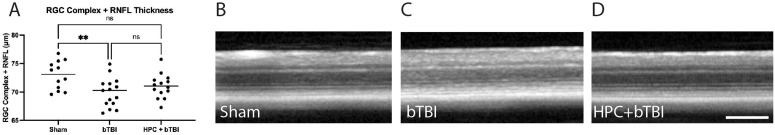
The RGC Complex + RNFL thickness decreases following bTBI. Structural analysis of the retina using OCT showed a RGC Complex + RNFL thickness significantly decrease in bTBI mice compared to sham mice (A-C, p = 0.002). There was not a significant difference in the RGC complex + RNFL thickness between sham mice and bTBI+ HPC mice (p = 0.051). Additionally, there was not a significant difference between bTBI mice and bTBI+ HPC mice (p = 0.21). (* = p < 0.05). One-way ANOVA with Fisher’s LSD post-test. Scale Bar in D = 200 µm.

Structural analysis of the retina using OCT showed a RGC Complex + RNFL thickness of 72.95 ± 2.51 µm in sham mice (Fig 2A, 2B), which was significantly thicker than mice exposed to bTBI (69.91 ± 2.56 µm, p = 0.002, Fig 2A, 2C). There was not a significant difference between sham mice and bTBI+ HPC mice (71.04 ± 2.11 µm, p = 0.051, Fig 2A, 2D). Likewise, there was not a significant difference between bTBI mice and bTBI+ HPC mice (p = 0.21).

**Fig 3:**
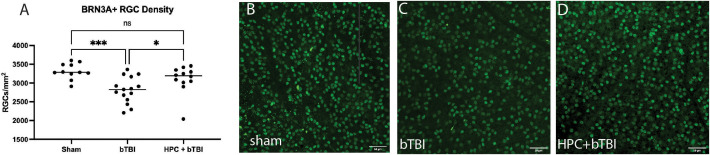
HPC preserves RGCs following blast. Analysis of BRN3A^+^ RGCs at the termination of the study showed that mice exposed to bTBI had significantly fewer surviving RGCs than sham mice (A-C, p = 0.0004). The number of surviving BRN3A^+^ RGCs in bTBI+ HPC mice (A, D) was not significantly different compared to sham mice (p = 0.12). However, there were significantly more BRN3A^+^ RGCs in HPC+ bTBI mice compared to bTBI mice (p = 0.02). (* = p < 0.05, *** = p < 0.001). One-way ANOVA with Fisher’s LSD post-test. Scale bar = 50 µm.

Analysis of surviving RGCs at the termination of the study showed 3317 ± 208.8 RGC/mm^2^ in sham mice (Fig 3A, 3B). Mice exposed to bTBI had significantly fewer surviving RGCs than sham mice (2811 ± 347.5 RGCs/mm^2^, p = 0.0004, Fig 3A, 3C). bTBI+ HPC mice (Fig 3A, 3D) had 3103 ± 375 RGCs/mm^2^, which was not significantly different compared to sham mice (p = 0.12), but was significantly more than bTBI mice (p = 0.02).

## Discussion

bTBI is recognized as a cause of visual dysfunction in military and Veteran populations, and multiple pre-clinical models show that the retina and optic nerve are vulnerable to damage and degeneration after blast exposure [1,2]. In rodent models a consistent phenotype has been that RGC dysfunction and loss have been observed following blast, with evidence that injury severity and cumulative blast exposure accelerate functional decline and cell loss [3–5,11]. In our present study we show that repetitive HPC delivered before blast shifts early post-injury outcomes toward preservation of RGC function and survival. Compared with bTBI-only mice, HPC-treated animals exhibited preserved PERG amplitude and significantly greater BRN3A^+^ RGC density four weeks following bTBI, with OCT outcomes trending toward preservation.

These results support the broader concept that bTBI-mediated visual deficits are shaped not only by the initial blast exposure, but also secondary cascades that evolve over time. These secondary processes can include neuroinflammation, activation of the immune system, and metabolic stress [3–5,7,9,20]. Prior work demonstrating that blast results in inflammation and immune activation has shown that immune modulation can improve visual outcomes, reenforcing the idea that post-bTBI processes influence retinal fate [6,8,9,21,22]. Our findings have also extended our previous preconditioning work that showed repeated low-level blast exposures before a higher-intensity blast reduced RGC loss and altered the injury-associated molecular response [12]. Taken together, blast preconditioning and HPC in the current study support a framework in which distinct, sub-injurious conditioning stimuli can engage endogenous resilience programs that reduce the probability that early damage propagates into sustained dysfunction and neurodegeneration [12,14,19].

A plausible explanation to our findings is that repetitive HPC activates hypoxia-response programs that increase stress tolerance to the secondary effects of bTBI. HPC is closely associated with HIF biology. Under hypoxic conditions HIF-1α stabilization and dimerization with HIF-1β enable transcription of factors associated with ischemic or hypoxic tolerance [13,23,24]. In neural tissue HIF-associated signaling can activate effectors including erythropoietin (EPO), heme oxygenase-1 (HO-1), and vascular endothelial growth factor (VEGF) that work to promote cellular survival and homeostasis [18,25–28]. EPO signaling has been shown to protect RGCs following axotomy [26], and has been linked to tissue protection and mitochondrial support [29,30]. Repetitive HPC has been shown to result in long-term tolerance of insult involving HO-1, which has been widely associated with antioxidant and anti-inflammatory processes relevant to neurodegeneration [27,31]. Although we did not directly assay these intermediates in this study, the preservation of PERG and BRN3A+ RGCs are consistent with the hypothesis that HPC reduces downstream signaling cascades rather than preventing the initial physical damage associated with blast exposure.

The relative behavior of our chosen endpoints also informs interpretation of the effect of HPC. At four weeks post bTBI, HPC preserved the PERG response and improved the BRN3A+ RGC counts relative to sham injured mice, while OCT thickness showed a smaller separation. This may reflect the timing of structural remodeling as we have previously shown [21], and the fact that OCT-derived thickness integrates neuronal, axonal, and glial contributions that change asynchronously. Longer follow-up and complementary structural metrics may therefore reveal a clearer structural signature of protection.

Our data are consistent with a larger preconditioning literature demonstrating that controlled HPC can induce retinal tolerance and protect RGCs in models of ischemia and glaucoma [14–17,23]. Broadly, preconditioning has been associated with increased antioxidant capacity and improved mitochondrial stress tolerance. Hypoxic conditioning can increase superoxide dismutase in non-retinal tissues [32], and preconditioning can reduce susceptibility to mitochondrial permeability in other organs [33,34]. Attenuation of inflammatory signaling has been reported following HPC, including reduced NF-κB/NLRP3-related pathways and SIRT1-associated protection of neurovascular function [35,36]. While these studies are not retina specific, they provide candidates for defining which HPC responsive pathways are most relevant to HPC mediated protection of bTBI induced RGC damage.

This study has several limitations. We used one HPC regimen, one blast paradigm, and a single post-injury time point. In future studies it will be important to test durability and generalizability across bTBI conditions and longer intervals that better reflect chronic trajectories [1,10,11]. Furthermore, this study used only male mice. In future studies it will be important to evaluate the effects of HPC using both sexes of mice. In addition, we did not directly measure HIF targets, oxidative injury markers, mitochondrial stress responses, or immune cell states, so defining the mechanism of protection will be important. Finally, establishing the minimal effective dose of HPC will be important for translational relevance and for distinguishing adaptive conditioning from potentially maladaptive hypoxic stress. Overall, our findings demonstrate that repetitive HPC prior to blast preserves RGC function and improves RGC survival, and together with our prior blast preconditioning data support a model in which conditioning stimuli engage endogenous neuroprotection that blunt secondary degenerative cascades after bTBI.

## Supporting information

S1 Dataset Data dictionary for raw data used in this study. (XLSX)

S2 Dataset Raw data for PERG, OCT, and RGC counts. (XLSX)

## References

[1] Evans LP, Roghair AM, Gilkes NJ, Bassuk AG. Visual outcomes in experimental rodent models of blast-mediated traumatic brain injury. Front Mol Neurosci. 2021;14:659576. doi: 10.3389/fnmol.2021.659576 33935648 PMC8081965

[2] Hussain SF, Raza Z, Cash ATG, Zampieri T, Mazzoli RA, Kardon RH, et al. Traumatic brain injury and sight loss in military and veteran populations- a review. Mil Med Res. 2021;8(1):42. doi: 10.1186/s40779-021-00334-3 34315537 PMC8317328

[3] Mohan K, Kecova H, Hernandez-Merino E, Kardon RH, Harper MM. Retinal ganglion cell damage in an experimental rodent model of blast-mediated traumatic brain injury. Invest Ophthalmol Vis Sci. 2013;54(5):3440–50. doi: 10.1167/iovs.12-11522 23620426 PMC4597486

[4] DeMar J, Sharrow K, Hill M, Berman J, Oliver T, Long J. Effects of primary blast overpressure on retina and optic tract in rats. Front Neurol. 2016;7:59. doi: 10.3389/fneur.2016.00059 27199884 PMC4842954

[5] Bernardo-Colón A, Vest V, Cooper ML, Naguib SA, Calkins DJ, Rex TS. Progression and pathology of traumatic optic neuropathy from repeated primary blast exposure. Front Neurosci. 2019;13:719. doi: 10.3389/fnins.2019.00719 31354422 PMC6637732

[6] Evans LP, Woll AW, Wu S, Todd BP, Hehr N, Hedberg-Buenz A, et al. Modulation of post-traumatic immune response using the il-1 receptor antagonist anakinra for improved visual outcomes. J Neurotrauma. 2020;37(12):1463–80. doi: 10.1089/neu.2019.6725 32056479 PMC7249480

[7] Harper MM, Gramlich OW, Elwood BW, Boehme NA, Dutca LM, Kuehn MH. Immune responses in mice after blast-mediated traumatic brain injury TBI autonomously contribute to retinal ganglion cell dysfunction and death. Exp Eye Res. 2022;225:109272. doi: 10.1016/j.exer.2022.109272 36209837

[8] Hubbard WB, Vekaria HJ, Velmurugan GV, Kalimon OJ, Prajapati P, Brown E, et al. Mitochondrial dysfunction after repeated mild blast traumatic brain injury is attenuated by a mild mitochondrial uncoupling prodrug. J Neurotrauma. 2023;40(21–22):2396–409. doi: 10.1089/neu.2023.0102 37476976 PMC10653072

[9] Guilhaume-Correa F, Pickrell AM, VandeVord PJ. The imbalance of astrocytic mitochondrial dynamics following blast-induced traumatic brain injury. Biomedicines. 2023;11(2):329. doi: 10.3390/biomedicines11020329 36830865 PMC9953570

[10] Harper MM, Jones M-G, Gramlich OW, Elwood BW, Boehme NA, Gilfanov N, et al. Identification of immune cell subsets involved in retinal ganglion cell damage following blast exposure. Exp Eye Res. 2026;267:110961. doi: 10.1016/j.exer.2026.110961 41794317

[11] Harper MM, Boehme NA, Dutca L, Navarro V. Increasing the number and intensity of shock tube generated blast waves leads to earlier retinal ganglion cell dysfunction and regional cell death. Exp Eye Res. 2024;239:109754. doi: 10.1016/j.exer.2023.109754 38113955

[12] Harper MM, Woll AW, Evans LP, Delcau M, Akurathi A, Hedberg-Buenz A, et al. Blast preconditioning protects retinal ganglion cells and reveals targets for prevention of neurodegeneration following blast-mediated traumatic brian injury. Invest Ophthalmol Vis Sci. 2019;60(13):4159–70. doi: 10.1167/iovs.19-27565 31598627 PMC6785841

[13] Bergeron M, Gidday JM, Yu AY, Semenza GL, Ferriero DM, Sharp FR. Role of hypoxia-inducible factor-1 in hypoxia-induced ischemic tolerance in neonatal rat brain. Ann Neurol. 2000;48(3):285–96. doi: 10.1002/1531-8249(200009)48:3<285::aid-ana2>3.0.co;2-8 10976634

[14] Liu J, Gu Y, Guo M, Ji X. Neuroprotective effects and mechanisms of ischemic/hypoxic preconditioning on neurological diseases. CNS Neurosci Ther. 2021;27(8):869–82. doi: 10.1111/cns.13642 34237192 PMC8265941

[15] Zhu Y, Zhang L, Gidday JM. Role of hypoxia-inducible factor-1α in preconditioning-induced protection of retinal ganglion cells in glaucoma. Mol Vis. 2013;19:2360–72. 24319330 PMC3850973

[16] Zhu Y, Ohlemiller KK, McMahan BK, Gidday JM. Mouse models of retinal ischemic tolerance. Invest Ophthalmol Vis Sci. 2002;43(6):1903–11. 12036997

[17] Harman JC, Guidry JJ, Gidday JM. Intermittent hypoxia promotes functional neuroprotection from retinal ischemia in untreated first-generation offspring: proteomic mechanistic insights. Invest Ophthalmol Vis Sci. 2020;61(11):15. doi: 10.1167/iovs.61.11.15 32910134 PMC7488620

[18] Grimm C, Hermann DM, Bogdanova A, Hotop S, Kilic U, Wenzel A, et al. Neuroprotection by hypoxic preconditioning: HIF-1 and erythropoietin protect from retinal degeneration. Semin Cell Dev Biol. 2005;16(4–5):531–8. doi: 10.1016/j.semcdb.2005.03.004 16144690

[19] Stetler RA, Leak RK, Gan Y, Li P, Zhang F, Hu X, et al. Preconditioning provides neuroprotection in models of CNS disease: paradigms and clinical significance. Prog Neurobiol. 2014;114:58–83. doi: 10.1016/j.pneurobio.2013.11.005 24389580 PMC3937258

[20] Bernardo-Colón A, Vest V, Clark A, Cooper ML, Calkins DJ, Harrison FE, et al. Antioxidants prevent inflammation and preserve the optic projection and visual function in experimental neurotrauma. Cell Death Dis. 2018;9(11):1097. doi: 10.1038/s41419-018-1061-4 30367086 PMC6203845

[21] Dutca LM, Stasheff SF, Hedberg-Buenz A, Rudd DS, Batra N, Blodi FR, et al. Early detection of subclinical visual damage after blast-mediated TBI enables prevention of chronic visual deficit by treatment with P7C3-S243. Invest Ophthalmol Vis Sci. 2014;55(12):8330–41. doi: 10.1167/iovs.14-15468 25468886 PMC5102342

[22] Zou Y-Y, Kan EM, Lu J, Ng KC, Tan MH, Yao L, et al. Primary blast injury-induced lesions in the retina of adult rats. J Neuroinflammation. 2013;10:79. doi: 10.1186/1742-2094-10-79 23819902 PMC3707737

[23] Semenza GL. Regulation of oxygen homeostasis by hypoxia-inducible factor 1. Physiology (Bethesda). 2009;24:97–106. doi: 10.1152/physiol.00045.2008 19364912

[24] Jiang BH, Rue E, Wang GL, Roe R, Semenza GL. Dimerization, DNA binding, and transactivation properties of hypoxia-inducible factor 1. J Biol Chem. 1996;271(30):17771–8. 8663540 10.1074/jbc.271.30.17771

[25] Grimm C, Wenzel A, Groszer M, Mayser H, Seeliger M, Samardzija M, et al. HIF-1-induced erythropoietin in the hypoxic retina protects against light-induced retinal degeneration. Nat Med. 2002;8(7):718–24. doi: 10.1038/nm723 12068288

[26] Kilic U, Kilic E, Soliz J, Bassetti CI, Gassmann M, Hermann DM. Erythropoietin protects from axotomy-induced degeneration of retinal ganglion cells by activating ERK-1/-2. FASEB J. 2005;19(2):249–51. doi: 10.1096/fj.04-2493fje 15556972

[27] Zhu Y, Zhang Y, Ojwang BA, Brantley Jr MA, Gidday JM. Long-term tolerance to retinal ischemia by repetitive hypoxic preconditioning: role of HIF-1alpha and heme oxygenase-1. Invest Ophthalmol Vis Sci. 2007;48(4):1735–43. doi: 10.1167/iovs.06-1037 17389506

[28] Lee J-C, Tae H-J, Kim IH, Cho JH, Lee T-K, Park JH, et al. Roles of HIF-1α, VEGF, and NF-κB in ischemic preconditioning-mediated neuroprotection of hippocampal CA1 pyramidal neurons against a subsequent transient cerebral ischemia. Mol Neurobiol. 2017;54(9):6984–98. doi: 10.1007/s12035-016-0219-2 27785755

[29] Peng B, Kong G, Yang C, Ming Y. Erythropoietin and its derivatives: from tissue protection to immune regulation. Cell Death Dis. 2020;11(2):79. doi: 10.1038/s41419-020-2276-8 32015330 PMC6997384

[30] Jacobs RA, Aboouf MA, Koester-Hegmann C, Muttathukunnel P, Laouafa S, Arias-Reyes C, et al. Erythropoietin promotes hippocampal mitochondrial function and enhances cognition in mice. Commun Biol. 2021;4(1):938. doi: 10.1038/s42003-021-02465-8 34354241 PMC8342552

[31] Liu R, Yang J, Li Y, Xie J, Wang J. Heme oxygenase-1: the roles of both good and evil in neurodegenerative diseases. J Neurochem. 2023;167(3):347–61. doi: 10.1111/jnc.15969 37746863

[32] Chen C-F, Tsai S-Y, Ma M-C, Wu M-S. Hypoxic preconditioning enhances renal superoxide dismutase levels in rats. J Physiol. 2003;552(Pt 2):561–9. doi: 10.1113/jphysiol.2003.045559 14561837 PMC2343376

[33] Huang W-Y, Jou M-J, Peng T-I. Hypoxic preconditioning-induced mitochondrial protection is not disrupted in a cell model of mtDNA T8993G mutation-induced F1F0-ATP synthase defect: the role of mitochondrial permeability transition. Free Radic Biol Med. 2014;67:314–29. doi: 10.1016/j.freeradbiomed.2013.11.019 24291231

[34] Javadov SA, Clarke S, Das M, Griffiths EJ, Lim KHH, Halestrap AP. Ischaemic preconditioning inhibits opening of mitochondrial permeability transition pores in the reperfused rat heart. J Physiol. 2003;549(Pt 2):513–24. doi: 10.1113/jphysiol.2003.034231 12692185 PMC2342939

[35] Lu X, Zhan L, Chai G, Chen M, Sun W, Xu E. Hypoxic preconditioning attenuates neuroinflammation via inhibiting NF-κB/NLRP3 axis mediated by p-MLKL after transient global cerebral ischemia. Mol Neurobiol. 2024;61(2):1080–99. doi: 10.1007/s12035-023-03628-w 37682454

[36] Vellimana AK, Aum DJ, Diwan D, Clarke JV, Nelson JW, Lawrence M, et al. SIRT1 mediates hypoxic preconditioning induced attenuation of neurovascular dysfunction following subarachnoid hemorrhage. Exp Neurol. 2020;334:113484. doi: 10.1016/j.expneurol.2020.113484 33010255 PMC8908895

